# Evaluating Birth Outcomes From a Community-Based Pregnancy Support Program for Refugee Women in Georgia

**DOI:** 10.3389/fgwh.2021.655409

**Published:** 2021-06-17

**Authors:** Elizabeth A. Mosley, Michelle Pratt, Ghenet Besera, Lasha S. Clarke, Heidi Miller, Tracy Noland, Bridget Whaley, Jennifer Cochran, Amber Mack, Melinda Higgins

**Affiliations:** ^1^Georgia State University School of Public Health, Atlanta, GA, United States; ^2^Emory University Rollins School of Public Health, Atlanta, GA, United States; ^3^Emory Decatur Hospital, Decatur, GA, United States; ^4^Embrace Refugee Birth Support, Clarkston, GA, United States; ^5^Healthy Mothers, Healthy Babies Coalition of Georgia, Atlanta, GA, United States; ^6^Emory University Nell Hodgson Woodruff School of Nursing, Atlanta, GA, United States

**Keywords:** refugees, perinatal health, pregnancy support, birth outcomes, community-based research, doulas

## Abstract

Refugee women face numerous and unique barriers to sexual and reproductive healthcare and can experience worse pregnancy-related outcomes compared with U.S.-born and other immigrant women. Community-based, culturally tailored programs like Embrace Refugee Birth Support may improve refugee access to healthcare and health outcomes, but empirical study is needed to evaluate programmatic benefits. This community-engaged research study is led by the Georgia Doula Access Working Group, including a partnership between academic researchers, Emory Decatur Hospital nurses, and Embrace. We analyzed hospital clinical records (*N* = 9,136) from 2016 to 2018 to assess pregnancy-related outcomes of Embrace participants (*n* = 113) and a comparison group of women from the same community and racial/ethnic backgrounds (*n* = 9,023). We controlled for race, language, maternal age, parity, insurance status, preeclampsia, and diabetes. Embrace participation was significantly associated with 48% lower odds of labor induction (OR = 0.52, *p* = 0.025) and 65% higher odds of exclusive breastfeeding intentions (OR = 1.65, *p* = 0.028). Embrace showed positive but non-significant trends for reduced cesarean delivery (OR = 0.83, *p* = 0.411), higher full-term gestational age (OR = 1.49, *p* = 0.329), and reduced low birthweight (OR = 0.77, *p* = 0.55). We conclude that community-based, culturally tailored pregnancy support programs like Embrace can meet the complex needs of refugee women. Additionally, community-engaged, cross-sector research approaches could ensure the inclusion of both community and clinical perspectives in research design, implementation, and dissemination.

## Background

Refugee women resettled in the United States (U.S.) face numerous and unique barriers to sexual and reproductive healthcare, and can experience worse pregnancy-related outcomes than U.S.-born and other immigrant women (1–8). Historically, the U.S. had the largest refugee resettlement program in the world, with over 3 million refugees resettled there since 1975—half of whom were women (9, 10). After the 2016 presidential election, U.S. resettlement numbers dropped to a record low of only 22,000 refugees in 2018 (down from 85,000 in 2016) (11). In turn, federal funding for refugee resettlement has also been reduced, endangering refugee-centered programming efforts (12, 13). Approximately one-third of the U.S. refugee resettlement agencies have been forced to close due to budget shortfalls (13).

The antecedent life experiences of refugee women, combined with the challenges of a hostile sociopolitical environment in the U.S., are associated with poorer pregnancy-related outcomes. Compared with U.S.-born and non-refugee immigrant women, refugee women begin prenatal care later and have fewer prenatal visits during pregnancy due to socioeconomic and language barriers, stigma, culturally insensitive health services, and other challenges (1, 4–6, 8). Additionally, a number of studies have shown that refugee women have higher rates of labor induction and cesarean delivery (2, 14), higher risk of preterm birth (1, 2), lower birthweight babies (1, 3), and lower rates of exclusive breastfeeding (15, 16). Although, notably, some studies have shown inconsistent results—for example, well-documented advantages in birth outcomes for foreign-born Latina women relative to U.S.-born women (17) and other studies that found African refugee women have better pregnancy outcomes than U.S-born women. Further, previous studies have found associations between characteristics of refugee and migrant populations, including race/ethnicity or country of origin (14, 18–20), length of time in the host country (19), parity (3, 20), and maternal age (20) and their maternal and child-health outcomes. For example, in a systematic review and meta-analysis, researchers found cesarean birth rates were higher among Sub-Saharan African and South Asian migrants compared with non-migrant women, but Eastern European and Vietnamese migrants had lower rates compared with non-migrant women (20). In addition to considering factors established as having an association with maternal and child-health outcomes, the diverse backgrounds (e.g., country of origin and length of time in the host country) of refugees can influence maternal and child-health outcomes.

Considerable literature describes the independent and joint benefits for healthy mothers and infants of spontaneous (vs. induced), vaginal (vs. cesarean) delivery at 37 weeks gestation or more (i.e., full term). For instance, induction of labor is associated with greater risk of maternal post-partum hemorrhage relative to spontaneous labor, and with greater risk of fetal stress and respiratory illness (21). Labor induction is also associated with a significantly higher risk of cesarean delivery (22), which itself is linked to acute and chronic complications, including postpartum cardiac arrest (23). Additionally, the increased risk of morbidity and mortality among infants born preterm or early term is also well-known (24–26). Low birthweight (<2,500 g), which is often a consequence of preterm birth, confers a higher risk of infant mortality and morbidity (including cognitive deficits and motor delays) that can extend throughout the life course (27). Particularly for low birthweight infants, though the benefit may extend to infants of normal birthweight, evidence suggests that initiation of breastfeeding within the first day of life is associated with a significant reduction in the risk of neonatal mortality as compared with breastfeeding delayed for >24 h after birth (28).

Community- and evidence-based pregnancy support programs—ones that provide support for pregnant women throughout the duration of their pregnancy and the postpartum period through strategies such as doula support and group education—have the potential to provide support and improve connection to culturally appropriate healthcare, but there is a dearth of evidence on how these programs impact refugee birth outcomes (29). In a mixed-methods study of the Refugee Women's Health Clinic in Arizona, researchers documented widespread barriers to prenatal care and high approval of the specialized clinic among refugee women from diverse ethnic backgrounds but did not assess birth outcomes (30). Another study of community-based doulas in New York demonstrated that having a doula of the same ethnic background improved Burmese refugee women's self-advocacy during labor and delivery (31). However, this qualitative study did not measure associations with maternal and child health outcomes. Similarly, the participants in a qualitative study of community-based prenatal services for refugee women in Perth, Australia, reported improved social support, greater continuity of care, increased knowledge about pregnancy, greater confidence to ask questions, and more assistance with other life challenges such as transportation and language services (32). Finally, a study with Burmese refugees in Melbourne, Australia, showed that group-based prenatal care can help women feel more informed, prepared, and confident; improve social and emotional support; and build trusting relationships with healthcare providers (33). But it is still unclear how group-based prenatal care and childbirth education translate to improved birth outcomes.

To date, evidence on reproductive health disparities and interventions for refugee women is sparse and incomplete. As described above, most studies use qualitative or mixed methods to evaluate the process and experience of refugee pregnancy support programs. Much less is known about how these programs quantitatively impact birth outcomes on a large scale. Additional research is needed to assess programmatic effects on reproductive health outcomes for refugees in the U.S. This requires both adequate sample sizes and statistical methods that can account for the vast diversity of refugee groups.

The current study is a community-engaged, quantitative evaluation of maternal health outcomes, child health outcomes, and breastfeeding intentions among the participants of the Embrace Refugee Birth Support program in Clarkston, Georgia. Founded in 2010, Embrace Refugee Birth Support (34) is a comprehensive, culturally tailored pregnancy support program offered by the non-profit, refugee support organization Friends of Refugees. The Embrace participants receive 8 weeks of no-cost, evidence-based childbirth education classes taught in their language by community liaisons. They are also matched to an Embrace volunteer, who provides transportation to prenatal and postnatal visits, continuous support during labor and childbirth, and social connection. Embrace uses an evidence-based “Healthy Moms” curriculum (35) for pregnant refugee women that covers topics including prenatal health, newborn care, and how to communicate with providers. In our study, we analyze hospital clinical records to compare birth outcomes for the Embrace participants and women who did not participate in the program, controlling for relevant covariates. We ask the following:

- Do the Embrace participants have improved maternal health outcomes, including reduced labor induction, vaginal delivery compared with similar women who did not participate in Embrace?
- Do the Embrace participants have improved child health outcomes, including higher birthweight and gestational age at birth compared with similar women who did not participate in Embrace?
- Do the Embrace participants have greater likelihood of breastfeeding intentions compared with similar women who did not participate in Embrace?

## Methods

### Research Approach and Setting

In 2019, the Healthy Mothers, Healthy Babies Coalition of Georgia (HMHBGA)—a non-profit organization dedicated to local maternal and child health—convened the Georgia Doula Access Working Group (GADAWG) (36). The GADAWG mission is to improve access to full-spectrum doula services in Georgia, especially for marginalized groups facing the greatest barriers to high-quality maternal and child healthcare, including refugees, people of color, and low-income families. This study is a community-engaged, cross-sector collaboration between Embrace Refugee Birth Support, Georgia State University School of Public Health, Emory School of Public Health, Emory School of Nursing, and Emory Decatur Hospital overseen and supported by the GADAWG. The members of our research team represent each of the organizations involved as well as the refugee communities we serve.

It is important to note that Clarkston, Georgia is a uniquely diverse community near Atlanta. The town has resettled over 37,000 refugees in the past 25 years, and the current population of nearly 13,000 is over 31% foreign-born with representation from 150 countries, 760 ethnic groups, and 140 languages all in one square mile. Emory Decatur Hospital—located only 3 miles from Clarkston—is where the majority of the Embrace participants give birth. Inclusive of Clarkston, metro-Atlanta is the ninth largest metro area in the country at over 6 million residents and the fourth fastest growing (37). Its population is also diverse with 46% White, 34% Black, 11% Hispanic, and 6% Asian residents. Yet racial/ethnic and economic inequality persist: Atlanta has the highest income inequality in the country, 76% of Black children live in high-poverty neighborhoods compared to 6% of White children, and Black women are 3 times as likely as White women to die from pregnancy-related causes (38, 39).

All research activities were approved by the Emory University Institutional Review Board (IRB00109995). The researchers were trained in research ethics through the Collaborative Institutional Training Initiative, including human subjects protection.

### Data and Measures

To evaluate the Embrace program, we abstracted maternal clinical records from March 2016 to December 2018 at Emory Decatur Hospital. There were a total of 9,136 unique clinical records during this period. We sought to include all covariates related to refugee maternal and child health outcomes, but we were limited by the hospital medical records. In the end, we included the Embrace participation, race and language spoken (as a proxy for ethnicity), age, parity, insurance status (as a proxy for socioeconomic status), preeclampsia, and diabetes.

#### Predictors

##### Embrace Participation

We used Embrace's internal program dataset to identify the names, dates of birth, and delivery dates for the Embrace program participants during the same period. Those names were cross-matched with the Emory Decatur Hospital clinical records to identify the patients who had participated in the pregnancy support program. We created a new variable called “Embrace Participation” where the participants were designated 1, and the non-participants were designated 0. There were 113 Embrace participants in the sample.

##### Race and Language Spoken

We operationalized race/ethnicity as the patients' race and their primary language spoken at home—both demographics that were captured in the electronic medical record system. Race included White, Black, Asian, American Indian, Hawaiian/Pacific Islander, Hispanic, other, and unknown. The language variable included over 50 different languages, which we categorized into English, Burmese/Karen, African languages, Arabic/Egyptian/Aramaic, and other/unknown. Race and language were highly colinear, so we created a composite variable Race-Language with seven categories: White-non-Arabic, Black-African, Black-Other (reference because this was the largest subsample), Asian-Burmese/Karen, Spanish-speaking, Asian-Nepali, Asian-Other, Arabic/Egyptian/Aramaic-speaking, and other/unknown race.

##### Additional Covariates

Age was measured as a continuous variable in the electronic medical records. Parity—the number of pregnancies ending in live births, stillborns, and miscarriages—was measured as a count variable that included the current birth. There were 24 different insurance statuses, which we classified into these categories: public insurance (Medicaid/Medicare) or other (self-pay, private insurance, military insurance, veteran's insurance, or other). We also included underlying maternal health conditions that are associated with our pregnancy-related outcomes of interest—pre-eclampsia and diabetes. The variables were dichotomous, where having the condition was coded as 1 while not having the condition was coded as 0.

##### Zip Code

To consider environmental and neighborhood effects on maternal and child health, we controlled for the patient's zip code of residence. Over 300 zip codes were represented in the Emory Decatur database. We classified these into a categorical variable: Clarkston (reference), metro-Atlanta (coded as 2), Georgia outside of metro-Atlanta (coded as 3), and outside of Georgia (coded as 4).

#### Outcomes

##### Labor Induction

When patients are admitted to Labor and Delivery at Emory Decatur Hospital, the provider orders indicate whether (1) the patient was in spontaneous labor or (2) the patient was being induced (i.e., labor is started through medicine like Pitocin). In our dataset, spontaneous labor was coded as 0, and induction was coded as 1.

##### Cesarean Delivery

When a patient gives birth at Emory Decatur Hospital, the nurses record whether it was a vaginal delivery or a cesarean delivery (i.e., by surgical operation). In our dataset, we coded vaginal delivery as 0, and cesarean delivery as 1.

##### Gestational Age at Birth (and Full-Term)

The nurses also record the gestational age at birth, which is measured in weeks and days—either estimated since the last menstrual period of the patient or confirmed by ultrasound. In our dataset, we measured gestational age as a continuous variable in days. We also created a dichotomous variable for full-term gestational age, which is set at 259 days or 37 weeks (19). We coded all births before or at 259 as 0 (not full term), and then coded all births after 259 days as 1 (full term).

##### Birthweight (and Low Birthweight)

The electronic medical record also includes the birthweight of the baby in kilograms. In our analyses, we used birthweight as a continuous measure, and then also created a dichotomous variable for low birthweight, which is defined as <2.5 kg (19). We coded low birthweight as 1, and anything at or above 2.5 kg as 0.

##### Breastfeeding Plans

When patients are admitted to the Labor and Delivery Unit for birth, nurses ask how they plan to feed their babies. The feeding intention of the patient is recorded in the electronic medical record as exclusive breastfeeding, bottle feeding with formula, or a mixture of both. For this study, we coded exclusive breastfeeding as 1 and any other feeding plans as 0. Notably, this variable measures the patient's *intention* for breastfeeding, not the actual behavior.

### Analysis

For our analyses, we first looked at descriptive statistics for the Embrace participants and the comparison group. We then tested those differences, using the appropriate bivariate tests. For continuous predictors and outcomes (maternal age, gestational age in days, and birthweight in kilograms), we used *t*-tests. For categorical predictors and outcomes (race language, insurance, pre-eclampsia, diabetes, zip code, labor induction, cesarean, full term, low birthweight, and exclusive breastfeeding), we used chi-squared tests. For parity, which is a count variable, we used a bivariate Poisson regression.

We then conducted multivariate analyses to test for differences between the Embrace participants and the comparison group after controlling for covariates (race-language, maternal age, parity, insurance status, preeclampsia, diabetes, and zip code). For the continuous outcomes of gestational age at birth and birthweight, we used multiple linear regression models. For the dichotomous outcomes (labor induction, cesarean, low birthweight, full term, and exclusive breastfeeding), we used multiple logistic regression models. We then assessed marginal effects of Embrace participation, using adjusted predicted probabilities. We checked all assumptions for our regression models, including multicollinearity. We also conducted Wald tests to test the hypothesis that Embrace was associated with changes in the maternal and child health outcomes.

## Results

### Descriptive Statistics by Embrace Participation

#### Demographics and Predictors

We analyzed 9,136 unique clinical records from Emory Decatur Hospital (Table 1). The majority of the patients in the sample (62.2%) were Black and spoke a language other than Arabic, Spanish, or a Bantu African language—most often, English. On average, the patients in our study were 29 years old, had two prior births, and were on public insurance (65.8%). There was a small but important minority of women who had pre-eclampsia (6.9%) and diabetes (8.6%).

**Table 1:** Descriptive statistics of the Embrace participants and comparison group at Emory Decatur Hospital from 2016 to 2018.

**Descriptor**	**Comparison**		**Embrace**		**Total**		**Statistic**	***p*-value**
	***n***	**%**	***n***	**%**	***n***	**%**		
Total	9,023	98.76	113	1.24	9,136	100	–	–
Race and Language^***^							χ^2^_8_ = 598.30	<0.001
White Non-Arabic speaking	1,370	15.57	2	1.77	1,372	15.4	–	–
Black African language	307	3.49	37	32.74	344	3.86	–	–
Black-Other	5,537	62.94	5	4.42	5,542	62.2	–	–
Asian-Burmese/Karen	360	4.09	34	30.09	394	4.42	–	–
Spanish-speaking	325	3.69	0	0	325	3.65	–	–
Asian-Nepali	236	2.68	1	0.88	237	2.66	–	–
Asian-Other	367	4.17	12	10.62	379	4.25		
Arabic-Speaking	85	0.97	9	7.96	94	1.05	–	–
Other/Unknown race	210	2.39	13	11.5	223	2.5		
Maternal age	28.81 (M)	6.01 (SD)	28.89 (M)	6.20 (SD)	28.81 (M)	6.01 (SD)	*t*_9, 124_ = −0.16	0.88
Parity^***^	2.24 (M)	1.45 (SD)	3.24 (M)	2.20 (SD)	2.26 (M)	1.47 (SD)	*b* = 0.37	<0.001
Insurance Status^***^							χ^2^_1_ = 45.1	<0.001
Other Insurance/Self-Pay	3,120	34.59	5	4.42	3,125	34.21	–	–
Public insurance: medicaid/medicare	5,901	65.41	108	95.58	6,009	65.79	–	–
Pre-Eclampsia^†^	624	6.92	3	2.65	627	6.86	χ^2^_1_ = 3.16	0.075
Accucheck	773	8.57	12	10.62	785	8.59	χ^2^_1_ = 0.60	0.44
Residence zip code^***^							χ^2^_3_=355.30	<0.001
Clarkston, Georgia	937	10.39	75	66.37	1,012	11.08	–	–
Metro-Atlanta, Georgia	7,945	88.07	38	33.63	7,983	87.4	–	–
Other Georgia	62	0.69	0	0	62	0.68	–	–
Outside of Georgia	77	0.85	0	0	77	0.84	–	–

*The b_parity_ coefficient is from a bivariate Poisson regression due to the count distribution of the parity variable;*

† p < 0.01; *p < 0.05;**p < 0.01;

*** *p < 0.001*.

Out of the total sample, 113 (1.2%) patients had participated in the Embrace program and 9,023 (98.8%) patients were used for the comparison group. The two groups differed by race and language spoken (χ^2^_8_ = 598.3, *p* < 0.001), parity (b_Poisson_ = 0.4, *p* < 0.001), insurance (χ^2^_1_ = 45.1, *p* < 0.001, and zip codes (χ^2^_3_ = 355.3, *p* < 0.001). The Embrace participants were more likely to be Asian and speak Burmese/Karen or Black and speak African language. They were also more likely to have higher parity, public insurance, and live in Clarkston than the comparison group.

#### Pregnancy-Related Outcomes

There is evidence that the Embrace participants had better pregnancy-related outcomes than the comparison group (see Table 2). Labor was induced for 15.0% of the Embrace participants compared with 24.7% of the comparison group (*p* = 0.02). Twenty-six percent of the Embrace participants had cesarean deliveries compared with 33.8% of the comparison group (*p* = 0.07). Embrace babies had significantly higher gestational age at birth: 277 days compared with 272 days in the comparison group (*p* = 0.01), and nearly 94% of the Embrace babies were born full-term compared with 88.9% in the comparison group (*p* = 0.05). Only 5.3% of the Embrace babies were low birthweight compared with 10.1% in the comparison group (*p* = 0.09). Finally, 62.9% of the Embrace participants planned to exclusively breastfeed compared with 63% of the comparison group (*p* = 0.93).

**Table 2:** Bivariate statistics by Embrace participation/comparison group at Emory Decatur Hospital from 2016 to 2018.

**Outcome**	**Comparison**		**Embrace**		**Total**		**Statistic**	***p*-value**
	***n***	**%**	***n***	**%**	***n***	**%**		
Labor induction^*^	2,225	24.66	17	15.04	2,242	24.54	χ^2^_1_ = 5.57	0.02
Cesarean delivery^†^	3,041	33.76	29	25.66	3,070	33.66	χ^2^_1_ = 3.27	0.07
Gestational age (days)^*^	271.69 (M)	18.33 (SD)	276.54 (M)	10.02 (SD)	271.75 (M)	18.26 (SD)	*t*_9, 009_ = −2.76	0.01
Full term gestation^†^	7,929	87.88	106	93.81	8,035	87.95	χ^2^_1_ = 3.7	0.05
Birthweight (kilos)^†^	3.17 (M)	0.75 (SD)	3.30 (M)	0.49 (SD)	3.17 (M)	0.75 (SD)	*t*_8, 662_ = −1.82	0.07
Low birthweight^†^	909	10.07	6	5.31	915	10.02	χ^2^_1_ = 2.81	0.09
Exclusive breastfeeding	5,191	63.33	61	62.89	5,252	63.32	χ^2^_1_ = 0.01	0.93

† *p < 0.01*;

* p < 0.05; **p < 0.01; ***p < 0.001.

### Multivariate Analyses

#### Regression Models

Results from our multiple logistic regression models similarly show some significantly improved pregnancy-related outcomes for the Embrace participants. Table 3 includes our outcomes of interest: labor induction, cesarean, full-term gestation, low birthweight, and exclusive breastfeeding intentions. We have included the total number of observations (*n*) and the model's fit-test statistics (likelihood ratio, LR, and chi-square). All models were statistically significant (*p* < 0.001), indicating we can reject the null hypothesis that all predictors in the models are equal to zero.

**Table 3:** Multivariate analyses of pregnancy-related outcomes for the Embrace participants and the comparison group at Emory Decatur Hospital from 2016 to 2018.

**Variable**	Labor induced^*^				**Cesarean delivery**				**Full term gestation**				**Low birthweight**				Exclusive breastfeeding^*^			
	***n*** **=** **8,871**				***n*** **=** **8,860**				***n*** **=** **8,871**				***n*** **=** **8,871**				***n*** **=** **8,071**			
	**LR chi**^**2**^ **(17)** **=** **44.56**				**LR chi**^**2**^ **(17)** **=** **394.11**				**LR chi**^**2**^ **(17)** **=** **494.16**				**LR chi**^**2**^ **(17)** **=** **422.59**				**LR chi**^**2**^ **(17)** **=** **1,051.76**			
	***p*** **=** **≤0.001**				***p*** **=** **≤0.001**				***p*** **=** **≤0.001**				***p*** **=** **≤0.001**				***p*** **=** **≤0.001**			
	**OR**	***p***	**95% CI**		**OR**	***p***	**95% CI**		**OR**	***p***	**95%CI**		**OR**	***p***	**95% CI**		**OR**	***p***	**95% CI**	
Embrace participation	0.52	0.025	0.29	0.92	0.83	0.411	0.53	1.30	1.49	0.329	0.67	3.34	0.77	0.552	0.33	1.82	1.65	0.028	1.06	2.57
**Race and language (reference: black-english)**																				
White, non-Arabic	0.82	0.014	0.70	0.96	0.67	<0.001	0.57	0.77	1.69	<0.001	1.33	2.14	0.47	<0.001	0.35	0.62	3.60	<0.001	2.92	4.44
Black-African	0.65	0.003	0.48	0.87	0.89	0.392	0.69	1.15	1.67	0.019	1.09	2.56	0.61	0.041	0.38	0.98	0.99	0.931	0.76	1.28
Asian-Burmese	0.49	<0.001	0.36	0.67	0.58	<0.001	0.44	0.76	1.72	0.013	1.12	2.65	0.43	0.001	0.25	0.72	0.93	0.564	0.73	1.19
Any-Spanish	0.79	0.076	0.61	1.03	0.78	0.047	0.61	1.00	1.36	0.109	0.93	1.97	0.60	0.023	0.39	0.93	1.03	0.797	0.81	1.32
Asian-Nepali	0.69	0.043	0.48	0.99	1.05	0.73	0.78	1.41	2.20	0.009	1.22	3.96	0.79	0.37	0.48	1.32	0.58	<0.001	0.43	0.78
Asian-Other	0.81	0.118	0.63	1.05	0.77	0.03	0.61	0.97	1.06	0.746	0.75	1.49	1.05	0.803	0.73	1.50	1.14	0.292	0.89	1.47
Any, Arabic	1.10	0.699	0.69	1.74	1.09	0.715	0.70	1.68	1.89	0.12	0.85	4.20	0.93	0.834	0.45	1.89	1.11	0.646	0.71	1.74
Other/unknown race	0.61	0.007	0.43	0.88	0.89	0.434	0.66	1.19	1.36	0.214	0.84	2.19	0.66	0.137	0.38	1.14	1.59	0.005	1.15	2.20
Maternal age	1.04	<0.001	1.03	1.05	1.06	<0,001	1.05	1.07	1.01	0.123	1.00	1.02	0.99	0.077	0.97	1.00	1.04	<0.001	1.03	1.05
Parity	1.17	<0.001	1.13	1.22	0.91	<0,001	0.88	0.94	0.88	<0.001	0.84	0.92	1.05	0.057	1.00	1.11	0.77	<0.001	0.74	0.80
Medicaid insurance	1.13	0.058	1.00	1.28	1.23	0.001	1.09	1.38	1.05	0.528	0.89	1.25	1.20	0.054	1.00	1.44	0.49	<0.001	0.43	0.56
Pre-Eclampsia	1.63	<0.001	1.36	1.95	2.29	<0,001	1.93	2.72	0.17	<0.001	0.14	0.20	5.71	<0.001	4.73	6.89	0.91	0.328	0.75	1.10
Diabetes	1.36	<0.001	1.16	1.61	1.61	<0,001	1.37	1.88	0.55	<0.001	0.45	0.68	1.28	0.039	1.01	1.63	0.77	0.004	0.65	0.92
**Zipcode (reference: clarkston)**																				
Metro-Atlanta	1.02	0.878	0.83	1.24	0.97	0.769	0.81	1.16	0.90	0.448	0.68	1.19	1.18	0.276	0.88	1.60	0.96	0.660	0.80	1.15
Other Georgia	1.60	0.121	0.88	2.91	0.96	0.901	0.53	1.75	0.79	0.595	0.33	1.87	1.50	0.388	0.60	3.75	1.65	0.212	0.75	3.62
Outside Georgia	1.34	0.310	0.76	2.34	0.87	0.615	0.50	1.50	0.63	0.203	0.30	1.29	2.05	0.06	0.97	4.35	0.68	0.19	0.39	1.21

* *p < 0.05 for Embrace participation*.

Relative to the comparison group, the Embrace participants had 48% lower odds of labor induction (OR = 0.52, *p* = 0.025), and 65% higher odds of planning to breastfeed exclusively (OR = 1.65, *p* = 0.028). The first row corresponds with Embrace participation (our independent variable). Following rows correspond with our covariates: race-language, maternal age, parity, insurance status, preeclampsia, diabetes, and zip code. Other results, while not statistically significant, also showed trends in the positive direction. The Embrace participants had 17% lower odds of cesarean delivery (OR = 0.83, *p* = 0.411), 49% higher odds of full-term gestational age (OR = 1.49, *p* = 0.329), and 23% lower odds of low birthweight (OR = 0.77, *p* = 0.552). Not shown in Table 3, our results also showed improved but non-significant improvements in gestational age at birth and birthweight: a 2.26-day increase in gestational age at birth (*b* = 2.26, *p* = 0.204) and a 0.06 kg increase in birthweight (*b* = 0.06, *p* = 0.403).

**Figure 1:**
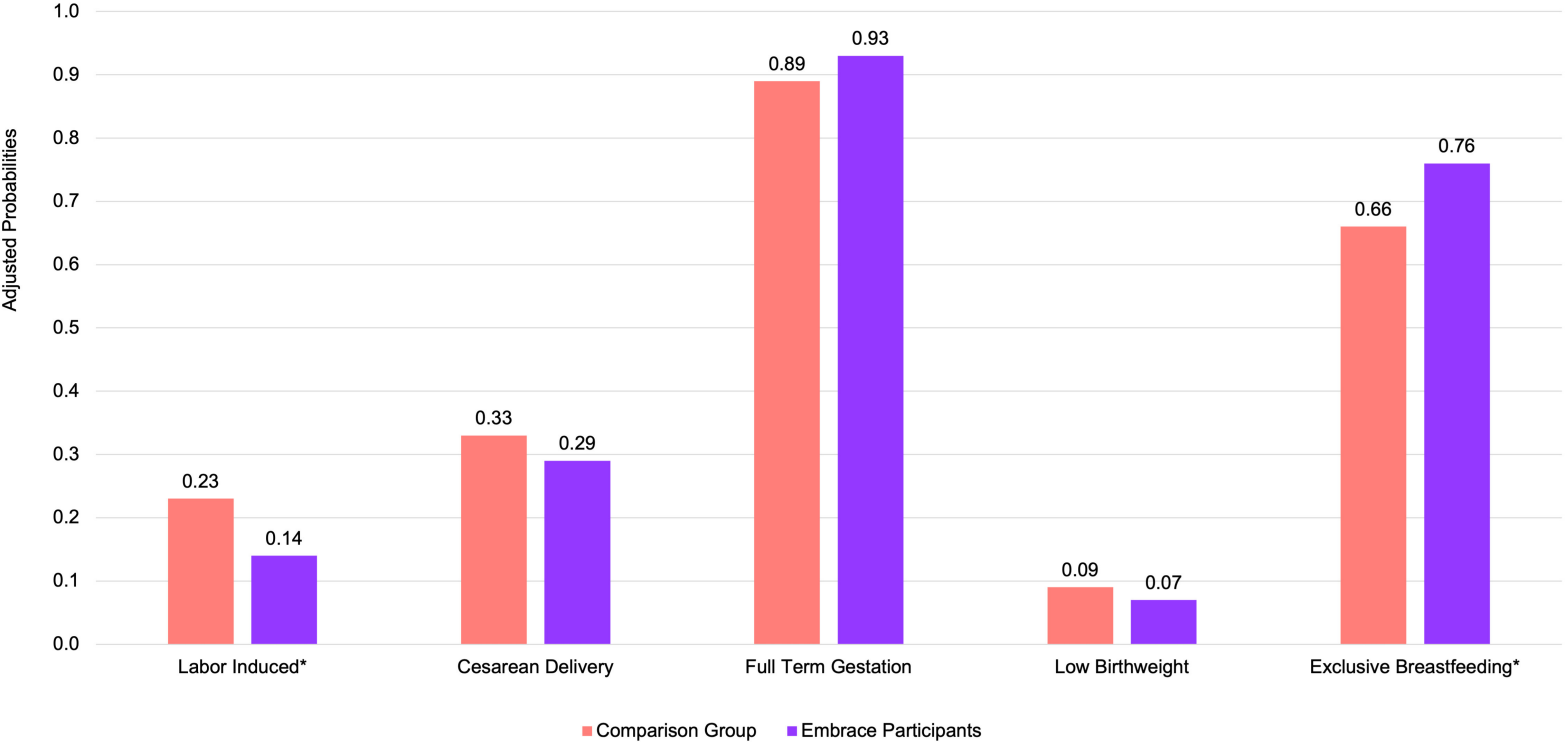
Marginal effects of Embrace participation on the probability of pregnancy-related outcomes at Emory Decatur Hospital from 2016 to 2018 after adjusting for covariates. *p < 0.05; probabilities are adjusted for race, language spoken at home, parity, maternal age, insurance status, underlying conditions (pre-eclampsia and diabetes), and zip code.

Holding other variables at their mean, we found that Embrace participation was significantly associated with a 9-percentage point reduction in labor induction from 23% (95% CI: 22.7–24.5%) to 14% (95% CI: 0.07–20.6%) and a 10-percentage point increase in exclusive breastfeeding plans (66 vs. 76%) (Figure 1). Our results also showed non-significant but positive trends on other outcomes. Embrace participation was associated with a 4-percentage point decrease in cesarean delivery from 33% (95% CI: 32.1–34.1%) to 29% (95% CI: 19.8–38.2%), a 4-percentage point increase in full-term gestational age from 89% (88.6–90%) to 93% (95% CI: 87.1–98.1%), and a 2-percentage point decrease in low birthweight from 9% (95% CI: 8.1–9.4%) to 7% (95% CI: 1.4–12.3%).

Our Wald tests indicate that we can reject the null hypothesis that Embrace participation was not associated with lower likelihood of labor induction (Wald test χ^2^_1_ = 5, *p* < 0.025) and greater likelihood of exclusive breastfeeding intentions (Wald test χ^2^_1_ = 4.83, *p* < 0.028). We cannot reject that null hypothesis for Embrace participation and cesarean delivery (Wald test χ^2^_1_ = 0.68, *p* = 0.411), full-term gestational age (Wald test χ^2^_1_ = 0.85, *p* = 0.329), or low birthweight (Wald test χ^2^_1_ = 0.35, *p* = 0.552).

## Discussion

Refugee women are particularly vulnerable to adverse pregnancy outcomes (2). Community-based pregnancy programs represent a promising strategy to address some of the challenges refugee women face when giving birth in a new country. Our study suggests that one such program, Embrace Refugee Birth Support in Georgia, might be effectively improving pregnancy-related outcomes for refugees, particularly reduced labor induction and increased plans for exclusive breastfeeding. Labor induction is, in turn, associated with higher gestational age and birthweight as well as a lower risk of cesarean delivery (22). While exclusive breastfeeding is associated with health benefits for mothers and babies across their life courses (28).

A core value of Embrace is cultural sensitivity; through their programming, education, and birth support, Embrace seeks to integrate a refugee's home culture into her birth experience in America. Additionally, the Embrace volunteers help alleviate many barriers women face in accessing and navigating pregnancy-related services, particularly as it relates to relationships and communication with providers during the pregnancy, birth, and postpartum periods (40). As highlighted by Khan and DeYoung (41), culturally sensitive programs and strategies such as these are needed to assist refugee women with accessing maternity services that can improve outcomes. Furthermore, prior studies have demonstrated that incorporating culturally sensitive strategies has been successful in addressing barriers, promoting perinatal health service use, and improving outcomes among pregnant women (29, 30). Findings from our study reinforce the importance of community-based, pregnancy support programs to improve refugee maternal and child health.

Given the benefits of breastfeeding to the health and development of infants, providing culturally relevant education on breastfeeding is important to improve breastfeeding-related outcomes among resettled refugee populations (15, 16). In Embrace's Healthy Babies and Breastfeeding module, community liaisons teach women about the health benefits of breastfeeding and navigating challenges with breastfeeding. While we are not able to directly assess the relationship in our study, previous studies have found women who attend prenatal classes focused on breastfeeding had higher intentions to exclusively breastfeed and were more likely to exclusively breastfeed, compared with women who did not attend classes (42, 43). This may explain the greater intention to exclusively breastfeed among the Embrace participants.

Education surrounding obstetrical interventions is critical for refugee populations, given many refugee women come from settings and cultures where such interventions are uncommon or unfamiliar, which may lead to avoiding medical care and distrust of providers (44–46). As found in another study, childbirth education may be an effective strategy for reducing elective inductions (47). Consistent with this finding, our study found that women who participated in Embrace had a significantly lower likelihood of labor induction, which, in turn, supports the findings that the women who participated in Embrace might have higher gestational age and birthweight as well as a lower risk of cesarean delivery. Furthermore, during many births, an Embrace volunteer is present with the moms to provide continuous support during labor and childbirth and to advocate for them and serve as a bridge between patient and provider, especially in instances where interventions are recommended. Our results align with findings from a systematic review that found women with continuous support during birth, including from doulas, were more likely to have a spontaneous vaginal delivery and less likely to have a Cesarean delivery (48). However, unlike our study, this review found that there was no impact of continuous support on the use of synthetic oxytocin (e.g., induction and augmentation) during labor or breastfeeding.

Findings from our study have several implications for providing services for pregnant refugee women and future research. Participating in culturally sensitive and tailored support services may have positive influences on refugee women's pregnancy outcomes and social support (19, 44). Similar programs can be developed to meet the pregnancy, birth, and postpartum needs of diverse resettled refugee populations. The Embrace participant sample was very diverse and can be used to generalize to many racial/ethnic groups. The Embrace sample was African (32.7%), Karen/Burmese (30.1%), other Asians who were not Nepali (10.6%), and Arab refugees (8.0%). Furthermore, our sample's diverse countries of origin are comparable to the resettled refugee population in the US. In Fiscal Year 2020, 35% of refugees resettling in the US came from Africa, 18% from East Asia, and 17% from Near East/South Asia (49). Therefore, our findings may be generalizable to the broader resettled refugee population in the US.

While the study findings align with Embrace's program components, future studies could assess Embrace's program evaluation data to discern possible mechanisms through which the program is improving pregnancy-related outcomes. In particular, this study was not able to assess how much doula support women received from Embrace or how many prenatal visits the Embrace and comparison women attended. Future research should include dose-response measures to assess those effects. Additionally, this was an innovative study, using big data to rigorously evaluate a community-based pregnancy support program for refugee women, whose health disparities are particularly difficult to quantify. Previous studies typically rely on qualitative designs or compare pregnancy outcomes between refugee and U.S.-born or immigrant populations. Future studies could adopt a similar design to quantitatively assess the effectiveness of services across resettled refugee populations. However, those studies will need to conduct careful power calculations to ensure the sample sizes are adequate to detect statistically significant results. Additionally, as found by several studies, the lack of cultural awareness and receptiveness among providers to refugee women's beliefs and practices surrounding birth creates disconnects between patients and providers and feelings of fear and marginalization among refugee women (41, 44, 50). In addition to birth education for refugee women, Embrace works closely and establishes relationships with healthcare providers in the community whom refugee women regularly see. Through these relationships, Embrace has worked to provide education, such as Lunch and Learns, for providers to orient them to the refugee community and different cultural practices. Future qualitative research is needed with data collection at the organizational and community levels to understand provider and community perspectives surrounding providing pregnancy-related services for refugee women.

### Limitations

Our study demonstrates the potential success of Embrace in pregnancy-related outcomes; however, important limitations must be noted. For one, women were not randomized into the Embrace program. Furthermore, causality cannot be established because we were unable to control for additional potential confounding factors or temporality. Nevertheless, we have controlled for a number of covariates that could have possibly explained the improved outcomes for the Embrace patients. Another related limitation is that we have no definitive data on the refugee status of women in the comparison group. We have made the comparison group as similar to the Embrace population as possible, but there could still be underlying unmeasured differences between the two groups. The use of secondary data from Emory Decatur Hospital limited which predictors and outcomes we could assess for this study. For example, data on length of time in the U.S. were not available for analysis. By using the hospital records, however, we were able to analyze a large sample, including the Embrace participants and a comparison group, using consistent, clinical measurements. Furthermore, due to limitations on data availability, only data on breastfeeding intentions were available rather than actual breastfeeding. Because behavior intentions are the closest predictor of actual behavior (51), however, this remains an important and valuable breastfeeding indicator. Finally, these results might not generalize to Spanish-speaking refugees in the US, given that there were no Spanish-speaking Embrace participants in this sample. Nevertheless, Embrace program evaluation data with the Spanish-speaking participants, who delivered at other hospitals, indicates the program is similarly valued by and beneficial for Latinas.

### Conclusion

Community-based, culturally tailored pregnancy support programs like Embrace are needed to meet the complex needs of refugee women, who are at greater risk of barriers to maternal health services and negative birth outcomes. In a time when anti-refugee social contexts and COVID-19 present additional challenges, programs such as Embrace are especially needed to support refugee women when navigating health services and giving birth in their new country. Community-engaged, cross-sector research approaches like the one we took in this study under the guidance of Georgia Doula Access Working Group are needed to ensure community and clinical perspectives are included in research design, implementation, and dissemination of results.

## Data Availability Statement

The data analyzed in this study is subject to the following licenses/restrictions: Data come from electronic medical records and cannot be made publicly available. Requests to access these datasets should be directed to emosley@gsu.edu.

## Ethics Statement

The studies involving human participants were reviewed and approved by Emory University IRB. Written informed consent for participation was not required for this study in accordance with the national legislation and the institutional requirements.

## Author Contributions

HM, TN, and JC: pregnancy support program development and implementation. EM, MP, HM, AM, and MH: study design. AM, HM, TN, and JC: community engagement. EM, MP, HM, TN, and JC: data collection. EM, MP, BW, and MH: data analysis. EM, GB, BW, and LC: first draft of manuscript, revisions, and second draft of manuscript. All authors contributed to the article and approved the submitted version.

## Conflict of Interest

The authors declare that the research was conducted in the absence of any commercial or financial relationships that could be construed as a potential conflict of interest.
